# Is the level of serum lactate dehydrogenase a potential biomarker for glucose monitoring with type 2 diabetes mellitus?

**DOI:** 10.3389/fendo.2022.1099805

**Published:** 2022-12-15

**Authors:** Yu-Shan Hsieh, Min-Chun Yeh, Yan-Yu Lin, Shuen-Fu Weng, Chung-Huei Hsu, Chen-Ling Huang, Yu-Pei Lin, A-Young Han

**Affiliations:** ^1^ Departmant of Nursing, School of Nursing, National Taipei University of Nursing and Health Sciences, Taipei, Taiwan; ^2^ Division of Endocrinology and Metabolism, Department of Internal Medicine, Taipei Medical University Hospital, Taipei, Taiwan; ^3^ Division of Endocrinology and Metabolism, Department of Internal Medicine, School of Medicine, College of Medicine, Taipei Medical University, Taipei, Taiwan; ^4^ Department of Nursing, College of Life Science and Industry, Sunchon National University, Suncheon, Republic of Korea

**Keywords:** glycemic variability, glucose monitoring, lactate dehydrogenase (LDH), glycated albumin (GA), type 2 diabetes mellitus

## Abstract

**Introduction:**

Type 2 diabetes mellitus (T2DM) is a metabolic disorder due to defects in insulin secretion or insulin resistance leading to the dysfunction and damage of various organs. To improve the clinical evaluation of short-term blood glycemic variability monitoring, it is critical to identify another blood cell status and nutritional status biomarker that is less susceptible to interference. This study identifies the significance of serum lactate dehydrogenase (LDH) level among T2DM patients treated in outpatient clinics and investigates the relationship of LDH level with other variables.

**Methods:**

This study comprised 72 outpatients with T2DM over 20 years of age. Blood samples were collected followed by a hematological analysis of serum glycated albumin (GA), LDH, fasting blood glucose, glycosylated hemoglobin, C-peptide, and insulin antibodies (insulin Ab).

**Results:**

Serum LDH level was significantly correlated with GA (p < 0.001), C-peptide (p = 0.04), insulin Ab (p = 0.03), and thyroid-stimulating hormone (TSH) levels (p = 0.04). Hence, we performed a linear regression analysis of hematological markers. GA (p < 0.001, r^2^ = 0.45) and insulin Ab (p < 0.001, r^2^ = 0.40) were significantly associated with LDH level. Then, we classified patients into low (<200 U/L) and high (≥200 U/L) serum LDH level groups, respectively. GA (p < 0.001), C-peptide (p = 0.001), and TSH (p = 0.03) showed significant differences in patients with high LDH levels compared with those in patients with low LDH levels.

**Conclusion:**

In conclusion, we suggested that LDH level was independent of long-term but associated with short-term blood glucose monitoring. The results indicated that changes in serum GA induced cell damage and the abnormal elevation of the serum level of LDH may occur simultaneously with glycemic variability. It has been reported that many biomarkers are being used to observe glucose variability in T2DM. However, LDH could provide a more convenient and faster evaluation of glycemic variability in T2DM.

## 1 Introduction

Type 2 diabetes mellitus (T2DM) is a metabolic disorder due to defects in insulin secretion or insulin resistance leading to the dysfunction and damage of various organs. To date, many hematological markers can be used to monitor the disease progression of T2DM, such as serum glycated albumin (GA), glucose AC (fasting blood glucose), glycosylated hemoglobin (HbA1c), connecting peptide (C-peptide), and insulin antibodies (insulin Ab). HbA1c is the most widely used indicator to determine the average glucose plasma concentration over the past 2 to 3 months prior to checkup in poorly controlled diabetes mellitus (DM). However, the HbA1c level in patients is significantly influenced by changes in the lifespan of erythrocytes (1). Clinicians may be falsely comforted by relatively low HbA1c values despite a high risk for other complications.

Serum GA is not affected by changes in the lifespan of erythrocytes in patients with T2DM with hemoglobinopathy. Serum GA measures the glycation of serum albumin, which is a protein with a half-life of approximately 14 days, and is an intermediate measure between HbA1c and serum blood glucose (2). GA level has been regarded as a specific inflammatory mediator related to the expression of inflammatory cytokines in muscle cells (3) and bovine retinal capillary pericytes (4), airway epithelial cells (5), and pancreatic β cells (6). Pu et al. observed that serum GA and C‐reactive protein (CRP) levels were significantly elevated and correlated in patients with T2DM and cardiac disease (7). Although GA level is not influenced by disorders of hemoglobin metabolism, it is affected by disorders of albumin metabolism and nutritional status.

Clinically, it is important to monitor the patient’s glycemic variability in T2DM. However, a single-spot blood glucose level is easily affected by food intake. GA is not routinely tested because it will be affected by albumin, and HbA1c is also easy to be influenced due to anemia or chronic disease history. Hence, to improve the clinical evaluation of short-term blood glycemic variability, it is critical to identify another biomarker that is less susceptible to interference.

Lactate dehydrogenase (LDH) is a critical marker in the metabolic pathway and also an intermediate metabolite that affects metabolism. It is often used to diagnose myocardial infarction, vessel damage, tissue injury, and certain types of malignant tumors (8). LDH may play a role in catalyzing pyruvate conversion into lactic acid through glycolysis. In DM, insulin is released according to glucose level, and it controls the metabolism of glucose through glycolysis followed by the oxidation of pyruvate in the mitochondria (9). Dmour et al. also observed that serum LDH level increased in association with serum glucose level (10). Although serum LDH level is reportedly elevated in patients with DM, the physiological effects remain unclear. This study identifies the significance of serum LDH level among T2DM patients treated in outpatient clinics and investigates the relationships of LDH level with other variables. The findings will aid in the formulation of treatment and preventive strategies for diabetes.

## 2 Materials and methods

### 2.1 Patients

**Figure 1:**
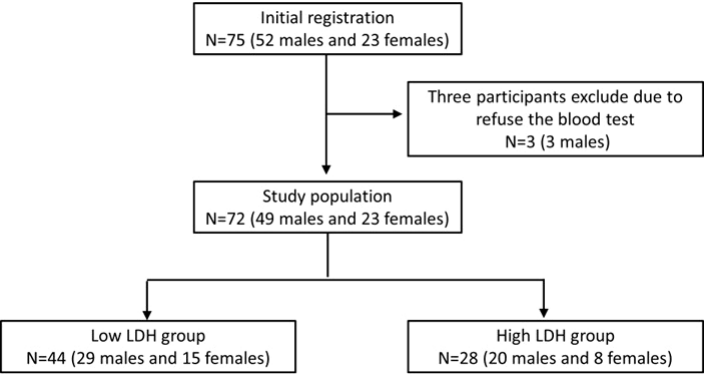
Flowchart showing the inclusion of the study population.

All participants were Taiwanese and were followed at the Taipei Medical University Hospital. The study protocol was approved by the Ethics Committee of the Institutional Review Board of Taipei Medical University. The procedures accorded with the ethical standards of the responsible committee on human experimentation (institutional and national) and the Helsinki Declaration. All participants provided informed consent to participate in the study. The study group comprised 72 participants (**Figure 1**) with T2DM (ICD-10-CM E11.9) over 20 years of age. Women who were pregnant, those under 20 years, and patients with neoplasms (ICD-10-CM C00-D49), cardiac disease (ICD-10-CM I00-I99), and chronic hepatitis or liver disease (ICD-10-CM K70-K77) were excluded from the study. The other exclusion criteria were diabetic complications (diabetic ketoacidosis or hyperglycemic hyperosmolar state), creatinine clearance <30 ml/min, age under 20 years, and pregnancy. For further analysis, patients were divided into low LDH level (<200 U/L, *n* = 44) and high LDH level (≥200 U/L, *n* = 28) groups and were compared using an independent ***t***-test.

### 2.2 Hematological analysis

Blood samples were collected from the participants after an 8-h fast. Hematological analysis of the level of serum glycated albumin (GA), lactate dehydrogenase (LDH), glucose AC (fasting blood glucose), glycosylated hemoglobin (HbA1c), connecting peptide (C-peptide), insulin antibodies (insulin Ab), triiodothyronine (T3), free thyroxine (FT4), and thyroid-stimulating hormone (TSH) was conducted. The hemoglobin level was determined using an automatic analyzer (XN-9000, Sysmex, Japan). The serum GA and LDH levels were analyzed by enzymatic methods by an automatic analyzer (ADVIA Chemistry XPT, Siemens, Germany), and shaking should be avoided while drawing blood. Glucose AC level was analyzed using the hexokinase method, and HbA1c level was determined through high-performance liquid chromatography using automatic analyzers (ADVIA Chemistry XPT and Variant II Turbo 2.0 System, Bio-Rad, USA). C-peptide level was detected by the method of chemiluminescence enzyme immunoassay using an automatic analyzer (ADVIA Chemistry XPT, Siemens, Germany). Serum insulin Ab level was measured by immunoradiometric binding assay in an automatic analyzer (PerkinElmer Cisbio, USA). The thyroid function profile (including T3, FT4, and TSH) was measured by the radioimmunoassay technique using an automatic analyzer (Atellica IM 1300 analyzer, Siemens, Germany).

### 2.3 Homeostasis model assessment of insulin resistance index

The modified homeostasis model assessment of insulin resistance (HOMA-IR) index is a simple and effective method for evaluating insulin sensitivity. The modified HOMA-IR was calculated using the following equation (11): 1.5 + (fasting blood glucose) × (fasting C-peptide)/2,800.

### 2.4 Statistical analysis

Results are presented as the value of mean and median. The independent *t*-test was used for continuous variables and the chi-square test for categorical variables. Pearson correlational analysis was applied to assess the correlations of serum LDH levels with other diabetes-related hematological markers. In addition, linear regression analysis was also performed to demonstrate the predictor of serum LDH levels. Statistical significance was indicated at *p <*0.05. Statistical analysis was conducted using IBM SPSS Statistics 22 (IBM Corporation, Somers, NY, USA) and MedCalc 20.113 (Med Calc Software Ltd., Ostend, Belgium).

## 3 Results

### 3.1 Participants and characteristics

As shown in **Table 1**, the demographic or metabolic characteristics did not differ between the two groups, including the number of patients using insulin and those taking oral medications. The duration of T2DM was 65.7 and 68.0 months, which also did not differ between the two groups. The use of antihyperglycemic agents and insulin in the two groups at baseline was similar.

**Table 1:** Clinical characteristics of the patients with T2DM. Data points are expressed in terms of the mean.

		Total	Low LDH	High LDH	*p*
**BMI [Mean(Median)]**		30 (27.9)	30.1 (27.6)	29.5 (27.7)	0.79
**Gender [Male N (%)]**		49 (68)	29 (65)	20 (71)	0.08
**Age [Mean(Median)]**		55.4 (15)	58.7 (58)	48.8 (48)	0.09
**Duration [Mean Month (Median)]**		65.9 (30.5)	65.7 (74.6)	68.0 (72.9)	0.76
**Antihypertensive agents [N(%)]**					
	Valsartan	3 (4.2)	1 (1.4)	2 (2.8)	0.70
	beta-blocker	2 (2.8)	1 (1.4)	1 (1.4)	0.60
	Amlodipine	3 (4.2)	2 (2.8)	1 (1.4)	0.54
**Antiplatelet agents [N(%)]**					
	Aspirin	12 (16.6)	6 (8.3)	6 (8.3)	0.22
**Antihyperglycemic agents [N(%)]**					
	Metformin	69 (95.8)	32(44.4)	37(51.4)	0.11
	Glimepride	11 (15.3)	6 (8.3)	5 (6.9)	0.42
	Linagliptin	18 (25)	9 (12.5)	9 (12.5)	0.54
	Dapagliflozin	11 (15.3)	6 (8.3)	5 (6.9)	0.54
	Saxagliptin and dapagliflozin	5 (6.9)	3 (4.2)	2 (2.8)	0.38
	Empagliflozin and Linagliptin	3 (4.2)	2 (2.8)	1 (1.4)	0.12
	Dulaglutide	4 (5.6)	2 (2.8)	2 (2.8)	0.60
**Insulin [N(%)]**					
	Short-acting insulin	4 (5.6)	2 (2.8)	2 (2.8)	0.70
	Insulin degludec	8 (13.2)	4 (5.6)	4 (5.6)	0.21
	Insulin glargine	4 (5.6)	2 (2.8)	2 (2.8)	0.24

### 3.2 The correlation of serum LDH levels with other diabetes-related hematological markers

**Table 2** presents the statistically significant correlations of serum LDH level with hematological markers. Serum LDH level was significantly correlated with GA (*p* < 0.001), C-peptide (*p* = 0.04), insulin Ab (*p* = 0.03), and TSH (*p* = 0.04) levels. However, serum LDH level was not significantly correlated with glucose AC (*p* = 0.10), HbA1c (*p* = 0.64), and HOMA-IR (*p* = 0.45) levels.

**Table 2:** The correlation of serum LDH levels with other diabetes-related hematological markers.

		LDH	Glucose AC	GA	HbA1c	C-peptide	HOMA-IR	Insulin Ab	T3	FT4	TSH
LDH	Correlation	*–*	0.12	0.63^**^	0.06	−0.26	−0.10	0.27^*^	0.18	−0.03	−0.25
	*p*	*–*	0.31	>0.001***	0.64	0.04*	0.45	0.03*	0.14	0.98	0.04*
Glucose AC	Correlation	0.12	*–*	0.52^**^	0.48^**^	0.07	0.63^**^	−0.05	−0.01	0.07	−0.04
	*p*	0.31	*–*	>0.001***	>0.001***	0.59	>0.001***	0.69	0.93	0.54	0.72
GA	Correlation	0.63^**^	0.52^**^	*–*	0.44^**^	−0.35	0.08	0.38^**^	−0.03	−0.08	0.05
	*p*	>0.001***	>0.001***	*–*	>0.001***	0.01*	0.57	0.01*	0.82	0.54	0.71
HbA1c	Correlation	0.06	0.48^**^	0.44^**^	*–*	−0.16	0.10	0.02	−0.116	−0.08	0.14
	*p*	0.64	>0.001***	>0.001***	*–*	0.24	0.44	0.86	0.36	0.51	0.27
C-peptide	Correlation	−0.26	0.07	−0.35	−0.16	*–*	0.77^**^		−0.021	−0.12	−0.11
	*p*	0.04*	0.59	0.01*	0.24	*–*	>0.001***		0.87	0.35	0.37
HOMA-IR	Correlation	-0.10	0.63^**^	0.08	0.10	0.77^**^	*–*	−0.01	0.03	0.12	−0.14
	*p*	0.45	>0.001***	0.57	0.44	>0.001***	*–*	0.94	0.82	0.34	0.27
Insulin Ab	Correlation	0.27^*^	−0.05	0.38^**^	0.02	0.01	−0.01	*–*	−0.17	−0.13	0.08
	*p*	0.03*	0.69	0.01*	0.86	0.97	0.94	*–*	0.18	0.32	0.52
T3	Correlation	0.18	−0.010	−0.03	−0.12	−0.02	0.03	−0.18	*–*	−0.011	−0.04
	*p*	0.14	0.93	0.82	0.36	0.87	0.82	0.18	*–*	0.93	0.77
T4	Correlation	−0.03	0.07	−0.08	−0.08	−0.12	0.12	−0.131	−0.01	*–*	−0.11
	*p*	0.98	0.54	0.54	0.51	0.35	0.34	0.32	0.93	*–*	0.39
TSH	Correlation	0.25*	−0.04	0.05	0.14	−0.11	−0.14	0.08	−0.04	−0.11	*–*
	*p*	0.040*	0.72	0.71	0.27	0.37	0.27	0.52	0.77	0.39	*–*

LDH, lactate dehydrogenase; GA, glycated albumin; glucose AC, fasting blood glucose, HbA1c, glycosylated hemoglobin; C-peptide, connecting peptide; HOMA-IR, homeostasis model assessment of insulin resistance index; insulin Ab, insulin antibodies; T3, triiodothyronine; FT4, free thyroxine; TSH, thyroid-stimulating hormone.

*p < 0.05; **p < 0.01; ***p < 0.001.

### 3.3 Regression analysis of the revealed markers and serum LDH association

**Figure 2:**
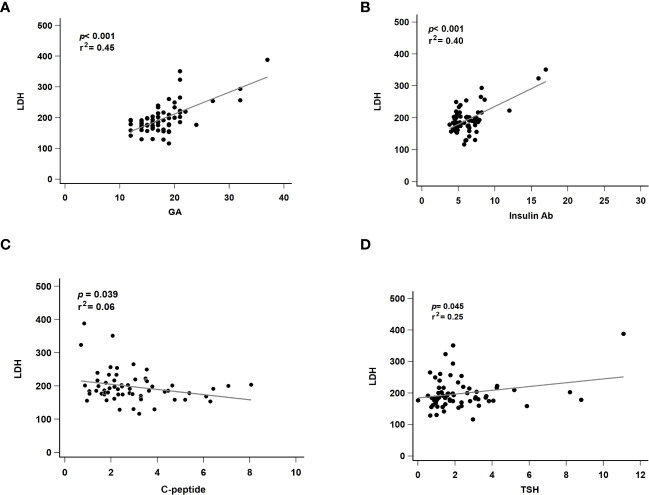
Regression analysis of revealed markers and serum LDH association. LDH, lactate dehydrogenase; GA, glycated albumin; C-peptide, connecting peptide; insulin Ab, insulin antibodies; TSH, thyroid-stimulating hormone. **(A)** The correlated with serum LDH and GA level. **(B)** The correlated with serum LDH and insulin Ab level. **(C)** The correlated with serum LDH and C-peptide level. **(D)** The correlated with serum LDH and TSH level. *p*, p-value; r^2^: r-squared.

To exclude the effects of liver disease, serum glutamate-pyruvate transaminase (GPT) level was also analyzed and found to be normal in all patients (mean: 26.5 U/L, data not shown). Next, we evaluated factors suspected to affect LDH levels. We performed a linear regression analysis of markers that were significantly correlated with serum LDH level. GA (*p* < 0.001, *r* ^2^ = 0.45, **Figure 2A**) and insulin Ab (*p* < 0.001, *r* ^2^ = 0.40, **Figure 2B**) were significantly associated with LDH level. Although C-peptide (*p* = 0.039, *r* ^2^ = 0.06, **Figure 2C**) and TSH (*p* = 0.045, *r* ^2^ = 0.25, **Figure 2D**) showed significant difference, their predictive ability was relatively insufficient.

### 3.4 The association of related markers between low levels and high levels of serum LDH

We divided participants into low and high serum LDH level groups, where low and high LDH levels were defined as normal (<200 U/L) and abnormal (≥200 U/L), respectively. The levels of GA (*p* < 0.001), C-peptide (*p* = 0.04), and TSH (*p* = 0.03) showed significant difference in patients with high LDH levels compared with those in patients with low LDH levels. Other markers were not significantly correlated with LDH level between the two groups (**Table 3**).

**Table 3:** The association of related markers between low (<200 U/L, *n* = 44) and high (≥200 U/L, *n* = 28) levels of serum LDH.

	Reference range	Total	Low LDH	High LDH	*p*
LDH	98–200 U/L	197.5 (186.0)	171.9 (176.0)	242.6 (222.0)	>0.001***
Glucose AC	<100 mg/dl	161.6 (144.5)	165.0 (147.0)	156.9 (134.0)	0.69
GA	11%–16%	19.8 (18.0)	17.9 (17.5)	21.9 (19.0)	0.001**
HbA1c	<5.5%	7.3 (6.8)	7.3 (6.9)	7.4 (6.9)	0.64
C-peptide	0.8–3.8 ng/ml	3.1 (2.6)	3.0 (2.8)	2.8 (2.3)	0.04*
HOMA-IR	<1.9	1.7 (1.6)	1.7 (1.6)	1.7 (1.6)	0.98
Insulin Ab	<7.5%	7.3 (6.0)	6.5 (5.8)	8.7 (6.1)	0.15
T3	60–181 ng/dl	95.2 (94.5)	94.1 (92.0)	97.0 (100.5)	0.05
FT4	0.74–1.47 ng/dl	1.2 (1.2)	1.2 (1.2)	1.4 (1.3)	0.80
TSH	0.5–4.7 μIU/ml	2.5 (1.6)	2.5 (1.9)	2.2 (1.4)	0.03*

LDH, lactate dehydrogenase; GA, glycated albumin; glucose AC, fasting blood glucose; HbA1c, glycosylated hemoglobin; C-peptide, connecting peptide; HOMA-IR, homeostasis model assessment of insulin resistance index; insulin Ab, insulin antibodies; T3, triiodothyronine; FT4, free thyroxine; TSH, thyroid-stimulating hormone.

*p < 0.05; **p < 0.01; ***p < 0.001.

### 3.5 Regression analysis of related markers in different serum levels of the LDH groups

**Figure 3:**
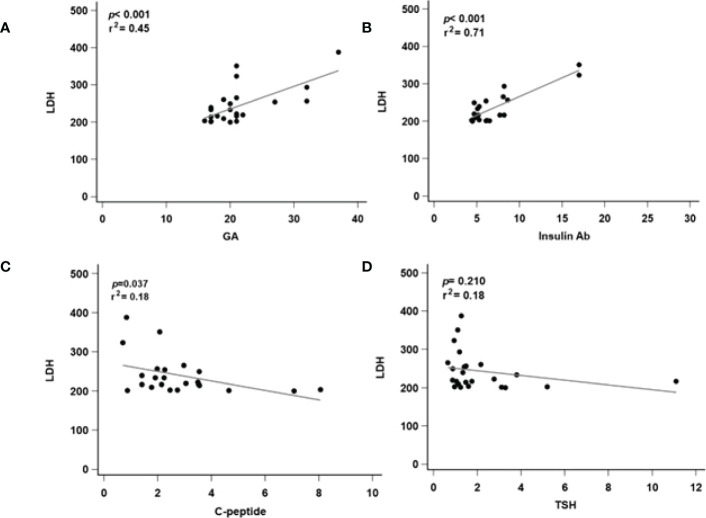
Regression analysis of related markers in high serum level of LDH groups. LDH, lactate dehydrogenase; GA, glycated albumin; C-peptide, connecting peptide; insulin Ab, insulin antibodies; TSH, thyroid-stimulating hormone. **(A)** The correlated with serum LDH and GA level. **(B)** The correlated with serum LDH and insulin Ab level. **(C)** The correlated with serum LDH and C-peptide level. **(D)** The correlated with serum LDH and TSH level. *p*, p-value; r^2^: r-squared.

**Figure 4:**
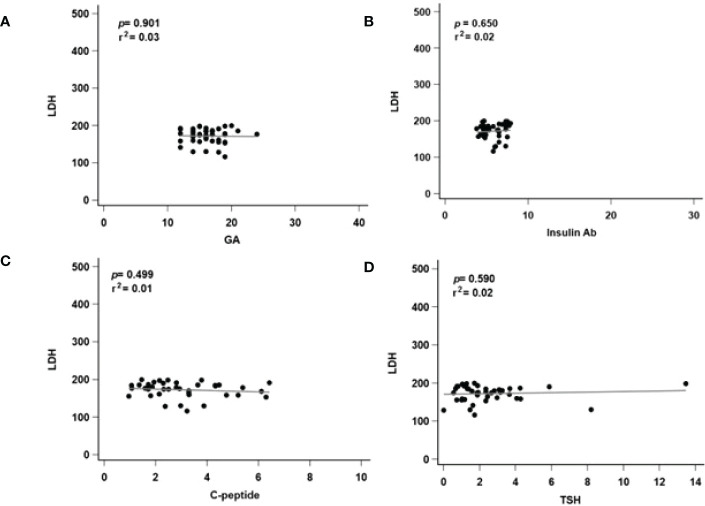
Regression analysis of related markers in low serum level of LDH groups. LDH, lactate dehydrogenase; GA, glycated albumin; C-peptide, connecting peptide; insulin Ab, insulin antibodies; TSH, thyroid-stimulating hormone. **(A)** The correlated with serum LDH and GA level. **(B)** The correlated with serum LDH and insulin Ab level. **(C)** The correlated with serum LDH and C-peptide level. **(D)** The correlated with serum LDH and TSH level. *p*, p-value; r*2*: r-squared.

To further check the correlation between different serum levels of the LDH groups, we further performed a linear regression analysis of markers that were significantly correlated with serum LDH level. In the high LDH group, GA (*p* < 0.001, *r* ^2^ = 0.45, **Figure 3A**) and insulin Ab (*p* < 0.001, *r* ^2^ = 0.71, **Figure 3B**) were significantly associated with LDH level. Although C-peptide (*p* = 0.037, *r* ^2^ = 0.018, **Figure 3C**) showed significant difference, its predictive ability was relatively insufficient. TSH (*p* = 0.210, *r* ^2^ = 0.18, **Figure 3D**) showed no significant difference. In the low LDH group, all of the biomarkers showed no significant difference in GA (*p* = 0.901, *r* ^2^ = 0.03, **Figure 4A**), insulin Ab (*p* = 0.650, *r* ^2^ = 0.02, **Figure 4B**), C-peptide (*p* = 0.499, *r* ^2^ = 0.01, **Figure 4C**), and TSH (*p* = 0.590, *r* ^2^ = 0.02, **Figure 4D**), and their predictive ability was relatively insufficient. These results might indicate that the correlation between LDH and GA as well as insulin Ab only exists when LDH is abnormal.

## 4 Discussion

**Figure 5:**
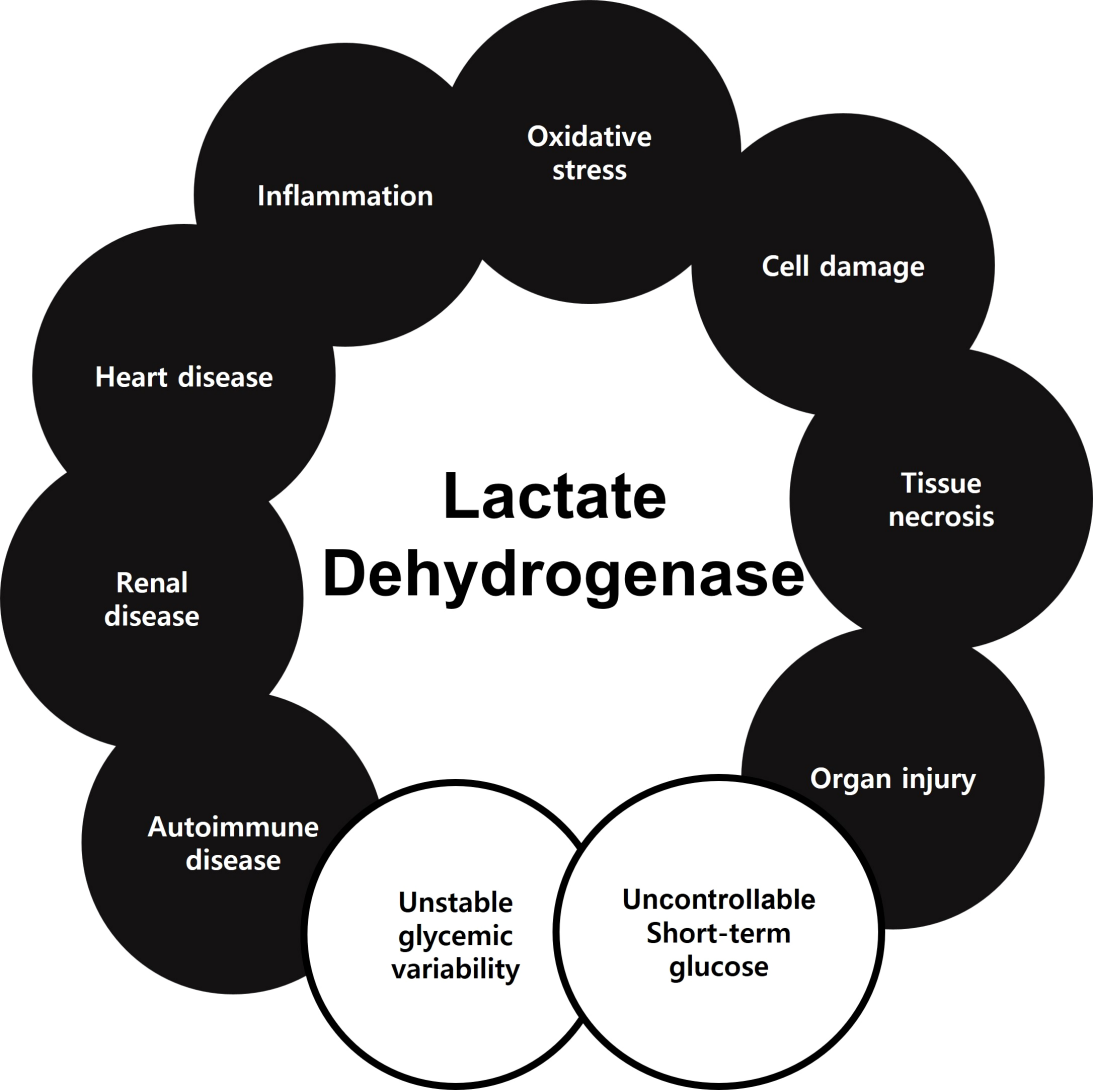
Previously studies showed that LDH could be a biomarker of oxidative stress, cell damage, tissue necrosis, organ injury, inflammation, heart disease, renal disease, and autoimmune disease. In the present study, LDH could serve as a biomarker of uncontrollable short-term glucose monitoring and unstable glycemic variability.

Serum LDH has been demonstrated to indicate many diseases or disorders. According to the results of this study, serum LDH level might also be an indicator of uncontrollable short-term glucose monitoring and unstable glycemic variability (**Figure 5**). Here, we demonstrated that a diabetic hematological marker, specifically abnormal elevated LDH, is associated with serum GA and insulin Ab levels.

Elevated LDH levels are observed in conditions such as tissue injury, necrosis, hypoxia, hemolysis, and severe inflammation (12). The overexpression of LDH activity has been known to interfere with normal glucose metabolism and insulin secretion due to increases in mitochondrial membrane potential, cytosolic free ATP, and cytosolic free Ca^2+^ in the islet beta cells (13). Oxidative modifications of albumin have been observed when the protein is glycated by pathophysiological concentrations of glucose in adipocytes. The glycation of albumin can be sufficient to oxidatively jeopardize adipocyte physiology and increase LDH activity in patients with diabetes (13). In a previous *in-vitro* study, LDH levels were demonstrated to increase in high-glucose treatments of INS-1 rat pancreatic beta cells (14). Another previous study in a population with coronavirus disease 2019 (COVID-19) also reported that fasting glucose at admission was positively associated with serum LDH level (15). In our results, serum LDH level was positively correlated with GA level, which may be due to oxidative modifications of albumin or expression of inflammatory cytokines in patients with T2DM. Therefore, we suggest that both LDH and GA might be affected simultaneously by hyperglycemia-related inflammation or oxidative stress.

To further evaluate the association between LDH and GA, we separated participants by serum LDH levels into two groups. Serum GA level was significantly elevated in patients in the high serum LDH level group than in patients in the low serum LDH level group. This suggested that serum LDH level might serve as a reference marker for short-term blood glucose monitoring, which is further supported by *in-vitro* findings (14).

Lactate metabolism and albumin levels may influence serum LDH and GA levels due to liver dysfunction; thus, we analyzed serum GPT levels to verify the measurements for all the patients who did not have liver dysfunction. In addition, diabetes and thyroid disorders have been reported to affect each other and to be associated with other conditions, and the thyroid hormone could also influence glycosylated HbA1c levels (16). In addition, serum glucose stabilization is also affected by thyroid function. Excess thyroid hormone stimulates lipolysis and the secretion of glucagon, followed by deterioration of glucose metabolism, which can cause glucose intolerance and insulin resistance in T2DM (17). To exclude variables that might have influenced the findings, we also examined thyroid function. However, in our study, T3, T4, or even TSH did not show a correlation or predictive ability with LDH. A possible reason could be that none of the patients in this study were diagnosed with thyroid disease; thus, the correlation with markers may be difficult to accurately observe under normal thyroid function status.

The administration of exogenous insulin for treatment usually induces insulin Ab to occur in the serum. The antibody might affect glycemic control in T2DM patients because of the binding insulin (18). A previous study indicated that patients with a high level of insulin Ab may develop severe clinical consequences, such as insulin resistance or hyperglycemia (19). An investigation by Zhu et al. shows that a higher level of insulin Ab was associated with increased daily glycemic variability in T2DM patients, indicating that patients with elevated insulin Ab should undergo glycemic monitoring for assessment (20). Another clinical study indicated that patients with a high serum level of insulin Ab have a longer duration of diabetes, a higher level of BMI, and a lower level of C-peptide (21). In our result, the higher level of LDH showed a correlation with insulin Ab. This might indicate that changes in serum markers may occur simultaneously with glycemic variability, followed by induction of cell injury and elevation of serum LDH level.

This study has several limitations. First, serum albumin level was not analyzed, which would have provided indications of nutritional status; it should be analyzed in future studies. Second, the study population was small in size. Although only a few patients were included in this study, the subjects were stringently selected to increase the reliability of the results. Third, although LDH levels were not related to the result of spot fasting glucose in our study, further examination of the relationship between LDH and continuous glucose monitoring still seems necessary. Fourth, because LDH levels were not related to the results of HbA1c and glucose AC, similar data might presumably be obtained from subjects without diabetes. Finally, we did not measure other inflammatory markers such as CRP that may have affected serum LDH levels. Therefore, the present study only demonstrated a significant correlation with LDH, GA, and insulin Ab levels, and a causal relationship should be explored in further studies.

## 5 Conclusions

In conclusion, we suggested that an elevated serum LDH level in patients with T2DM was associated with serum levels of GA and insulin Ab but not HbA1c. LDH level was independent of long-term but associated with short-term blood glucose monitoring. The result indicates that changes in serum GA elevation induced cell damage, and an abnormal serum LDH level may occur simultaneously with glycemic variability.

In clinical practice, LDH could be a low-cost, rapid, and easily measurable serum biomarker to evaluate short-term glycemic variability. It has been reported that many biomarkers are being used to observe glucose variability in T2DM. However, LDH could provide a more convenient and faster evaluation, especially as a biomarker for short-term glycemic variability monitoring. Further clinical and laboratory study is necessary to elucidate the underlying effect of inflammatory status on serum LDH and GA levels. The findings will aid in the formulation of treatment and preventive strategies for diabetes.

## Data availability statement

The original contributions presented in the study are included in the article/supplementary material. Further inquiries can be directed to the corresponding author.

## Ethics statement

The studies involving human participants were reviewed and approved by Institutional Review Board of Taipei Medical University. The patients/participants provided their written informed consent to participate in this study.

## Author contributions

Conceptualization: Y-SH and M-CY. Methodology: Y-SH. Software: Y-SH. Validation: M-CY, Y-YL and S-FW. Formal analysis: M-CY. Investigation: Y-SH. Resources: Y-SH. Data curation: M-CY. Writing—original draft preparation: Y-SH. Writing—review and editing: Y-SH and Y-YL. Visualization: Y-SH, M-CY, Y-YL, S-FW, C-HH, C-LH, Y-PL, and A-YH. All authors contributed to the article and approved the submitted version.
